# Engagement of non-governmental organisations in moving towards universal health coverage: a scoping review

**DOI:** 10.1186/s12992-021-00778-1

**Published:** 2021-11-16

**Authors:** Arman Sanadgol, Leila Doshmangir, Reza Majdzadeh, Vladimir Sergeevich Gordeev

**Affiliations:** 1 Department of Health Policy & Management, Tabriz Health Services Management Research Center, School of Management&Medical Informatics, Tabriz University of Medical Sciences, Tabriz, Iran; 2 Social Determinants of Health Research Center, Tabriz Univerisity of Medical Sciences, Tabriz, Iran; 3 CenterCommunity Based Participatory Research Center and Knowledge Utilization Research Center, Tehran Univerisity of Medical Sciences, Tehran, Iran; 4 Wolfson Institute of Population Health, Queen Mary University of London, London, UK; 5 Department of Infectious Disease Epidemiology, London School of Hygiene & Tropical Medicine, London, UK

**Keywords:** Non-governmental organisations, Universal health coverage, Health system, Health policy and systems research, Engagement strategy

## Abstract

**Background:**

Developing essential health services through non-governmental organisations (NGOs) is an important strategy for progressing towards Universal Health Coverage (UHC), especially in low- and middle-income countries. It is crucial to understand NGOs’ role in reaching UHC and the best way to engage them.

**Objective:**

This study reviewed the role of NGOs and their engagement strategies in progress toward UHC.

**Method:**

We systematically reviewed studies from five databases (PubMed, Web of Science (ISI), ProQuest, EMBASE and Scopus) that investigated NGOs interventions in public health-related activities. The quality of the selected studies was assessed using the mixed methods appraisal tool. PRISMA reporting guidelines were followed.

**Findings:**

Seventy-eight studies met the eligibility criteria. NGOs main activities related to service and population coverage and used different strategies to progress towards UHC. To ensure services coverage, NGOs provided adequate and competent human resources, necessary health equipment and facilities, and provided public health and health care services strategies. To achieve population coverage, they provided services to vulnerable groups through community participation. Most studies were conducted in middle-income countries. Overall, the quality of the reported evidence was good. The main funding sources of NGOs were self-financing and grants from the government, international organisations, and donors.

**Conclusion:**

NGOs can play a significant role in the country’s progress towards UHC along with the government and other key health players. The government should use strategies and interventions in supporting NGOs, accelerating their movement toward UHC.

**Supplementary Information:**

The online version contains supplementary material available at 10.1186/s12992-021-00778-1.

## Background

Health systems must be sufficiently efficient in population, service and financial coverage to achieve sustainable Universal Health Coverage (UHC) [1]. Much of the health policy debate currently focuses on achieving the 2030 Sustainable Development Agenda, essential for UHC. Progress towards UHC is one of the essential tools for improving health and well-being in the coming years [2]. According to WHO, UHC means that all people have access to the health services they need, when and where they need them, without financial hardship. It includes the full range of essential health services, from health promotion to prevention, treatment, rehabilitation, and palliative care [3].

However, despite injecting financial resources into health systems, many counties still face difficulties in progress towards UHC and did not provide preventive and curative health care services [4–9]. In many low- and middle-income countries (LMICs), the challenge of adequate provision of quality care to all who needs it becomes even more apparent, as all available human resources for health (both public and private) are required to achieve this goal. However, it is time to change how we look and think about health issues and health services provision if we want to achieve health attainment and well-being for all [2].

Given the inclusion of UHC in global health programs, new attention has been paid to heterogeneous groups of non-governmental organisations (NGOs) in health services provision and how they can help achieve public health goals. The term “NGO” usually refers to any non-profit voluntary group of global citizens who work locally, nationally and internationally for various cultural, social, charitable, and professional purposes [10]. Different alternative terms can describe NGOs, including voluntary, non-profit, grassroots organisations, and local groups. However, regardless of the term used, at the heart of civil society, NGOs are recognised as one of the most important and best tools for dealing with global issues such as the environment, peace and poverty [11, 12].

NGO’s role in the health sector has also changed in recent years, and significant emphasis has been placed on NGO contracts for service delivery [13]. In LMICs, NGOs play a significant role in financing and providing health care services, and the use of NGOs in advancing public health goals is increasingly common [14]. In some areas, NGOs seem to be the best tool for developing essential health services and are part of the strategy to achieve UHC [15]. NGOs are uniquely committed to providing health services in sparsely populated areas globally, mainly through their active participation in providing health services directly through the ancillary factors of supply [16]. Many governments partnered with NGOs, recognising their significant and often dominant role in providing health services in LMICs [15, 17–19]. Proponents of formal government interaction with NGOs argue that they operate extensively, even in remote and rural areas, and are more accountable than their public-sector counterparts [14]. Governments can also hold NGOs responsible for adhering to standards and achieving results, improving their service quality [20].

There are transparent and established links between governments and NGOs in many countries, while their relationship is ambiguous in others. This uncertainty can have adverse effects for both NGOs and governments, leading to service duplication and competition. Overall, there is mixed evidence regarding NGOs’ participation in health services provision in LMICs, their impact on the quality of service, and related direct out-of-pocket costs, ranging from positive to mild or weak effects [21–24]. Hence, this study aimed to review NGOs’ role and their engagement strategies in moving toward UHC.

## Methods

Our scoping review followed the Preferred Reporting Items for Systematic reviews and Meta-Analyses protocol, an extension for Scoping Reviews (PRISMA-ScR) (shown in Appendix S1) [25]. Our primary research question was “What is the role of NGOs in achieving the goals of UHC in various contexts?”. More specifically, we focused on the role of NGOs in moving towards UHC based on the three dimensions (population, service and financial coverage) and engagement strategies they used to achieve the goal.

### Inclusion and exclusion criteria

We included studies that investigated NGOs interventions in public health-related activities and UHC. The language of publications was restricted to English, and there was no time limit. Summaries, posters, letters to the editor, reviews, commentaries and opinion pieces were included in the review.

### Data sources and search

We searched five databases (PubMed, Web of Science (ISI), ProQuest, EMBASE and Scopus) from December 2019 to August 2020. To ensure the literature review’s comprehensiveness, we manually searched for references in the included articles. The complete search strategy can be found in Appendix S2.

### Study selection and data extraction

Results from the bibliographic databases were merged, and duplicates were removed. Two reviewers (LD and AS) independently screened the search results by title, abstract and full text. Disagreements were resolved by discussion and consensus. We extracted the following information from the studies included in the review, i.e., first author, country and date, study type and design, data collection method, quality appraisal, intervention(s), and NGOs’ role based on the UHC cube dimensions. Relevant information from retrieved articles was extracted for a narrative synthesis by both reviewers.

### Quality appraisal

The quality of the selected papers was assessed using the mixed methods appraisal tool (MMAT) [26]. The MMAT is effective as it is designed to appraise the most common types of empirical studies, including qualitative, quantitative and mixed-methods studies [27]. The MMAT is based on constructionist theory and has already been used by more than 100 systematic mixed study reviews. Two researchers (LD, AS) independently appraised the included studies using MMAT. The differences in the researchers’ appraisals were resolved by discussion (more details in Appendix S3).

### Synthesis of results

We synthesised results using directed content analysis and categorised findings based on the UHC cube’s dimensions (i.e., population, service and financial coverage). Identified items were coded into sub-themes using deductive reasoning. The primary categorisation of recommended codes was determined using available research concerning NGOs’ engagement in moving toward UHC.

## Results

### Selection of sources of evidence

**Fig. 1:**
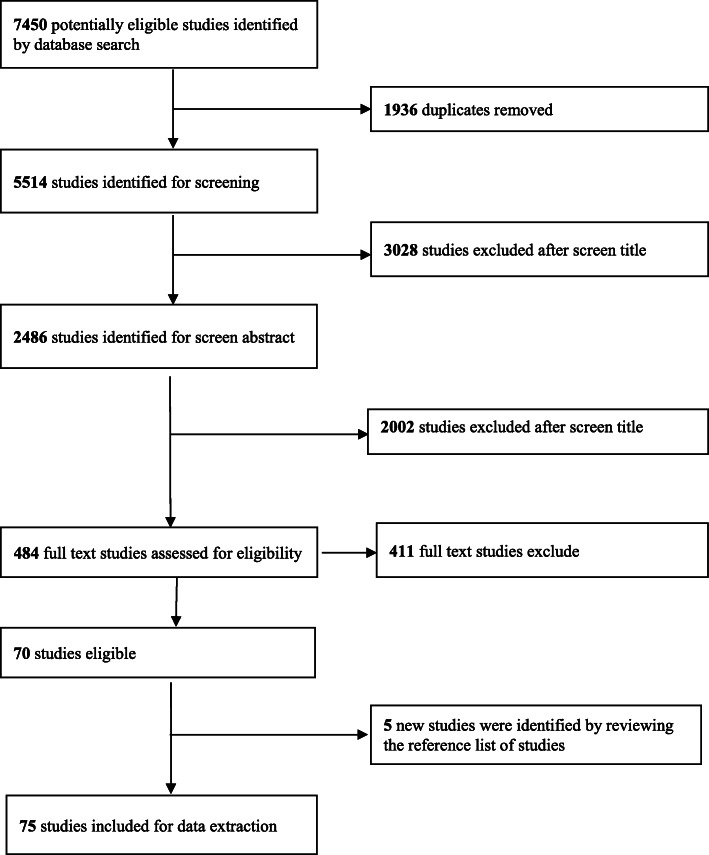
Study selection flow

The results of the screening process are shown in Fig. 1. Of 7540 studies, 5514 studies were screened by title and abstract for possible inclusion in the review. The full text of 484 articles was obtained and assessed for eligibility. An additional five studies were identified through manual search. Seventy-five studies met the eligibility criteria and were included in the final review. The main reasons for exclusion were a lack of focus in studies on relevant UHC dimensions, which focused on interventions not related to the health system.

### Characteristics of sources of evidence

Studies were conducted in 51 countries and five regions (Eastern and Central Europe, sub-Saharan Africa, South and Southeast Asia, the Caribbean and Latin America). Four studies were conducted in high-income countries, ten in upper-middle-income countries, 19 in LMICs, and 18 in low-income countries (Table 1).

**Table 1:** Study characteristics

First author (reference no)	Country, date and type	Study design	Data collection method	MMAT score	Intervention(s)
Yagub, A. I. [28]	Sudan, 2015/ LI	Mix method	Documentation/ observation, recordings and open interviews	*****	Providing health services for vulnerable people
Albis, M. L. F. [29]	Bangladesh, 2019/ LMI	Survey	Questionnaire	****	Providing primary health services to poor urban communities
Amirkhanian, Y. A. [30]	Central and Eastern Europe, 2004	Qualitative study	In-depth interviews	*****	Providing prevention, education and other services for people with AIDS
Bechange, S. [31]	Uganda, 2010/ LI	Mixed methods	Document review and analysis, observation, in-depth interviews, a structured questionnaire, and conceptual events	*****	Providing HIV / AIDS health services to people vulnerable to HIV / AIDS
Ejaz, I. [32]	Pakistan, 2011/ LMI	Qualitative study	Document review and in-depth interviews	*****	Providing health education, health promotion and health services to the community
Mercer, M. A. [33]	Timor-Leste, 2014/ LMI	Qualitative study	In-depth explanation	**	Providing health services to a traumatised population
Wamai, R. G [34]	Kenya and Ethiopia, 2008/ LMI, LI	Qualitative study	In-depth explanation	***	NGOs provide public and health services (treatment, prevention and rehabilitation), HIV/AIDS and reproductive health services to urban areas
Mercer, A [35]	Bangladesh, 2004/ LMI	Review	Review of report	***	Providing clinical education, vaccination, reproductive health and child health services to the poorest people
Mercer, A [36]	Bangladesh, 2006/ LMI	Survey	Structured interviews	****	Providing reproductive health services in rural areas among the poor and needy in 12 areas
De Maio, G. [37]	Italy, 2014/ HI	Descriptive study	Routine program data.	****	Providing inpatient and outpatient services to the homeless
De Souza, R. [38]	India, 2009/ LMI	Qualitative study	Analyse documents, in-depth interviews	*****	Organising sex workers in the slums of Bangalore, India, for HIV/AIDS prevention
Dhingra, R. [39]	India, 2001/ LMI	Qualitative study	N/A	**	Financing the provision of preventive and curative services through community-based health insurance programs for the Indian population
Franco, M. M. R. [40]	Mexico, 2019/ UMI	Quantitative retrospective analysis	Records	*****	Providing pharmaceutical and chemical subsidies to hematopoietic cell transplant patients
Gellert G. A [41]	1996	Qualitative study	Review of report	**	Providing medical services and prevention of tobacco-related diseases, infectious and epidemic diseases, maternal mortality and women’s health for the poorest people
Ghosh, S. C. [42]	Bangladesh, 2011/ LMI	Mix method	Pre-tested questionnaire and focus group discussions	*****	Establishing toilets in rural areas to improve their health.
Gilson, L. [43]	Ghana India Malawi Mexico Nepal Pakistan PNG South Africa Tanzania Uganda, 1994/ LMI, LI, UMI,	Narrative review	N/A	N/A	Providing health service, facilities and community participation
Gomez-Jauregui, J. [44]	Mexico, 2004/ UMI	Qualitative study	In-depth interview	*****	Providing reproductive health services for rural area
Heard, A [45]	India, 2011/ LMI	Mix method	Questionnaires/ interview and focus group discussion	*****	Providing essential health services in remote areas.
Holland, C. E. [46]	Cameroon, 2015/ LMI	Cross-sectional survey	Structurer questioner	*****	Providing AIDS/HIV services to NGOs for men who have sex with men (MSM)
Khan, J. A. [47]	Bangladesh, 2017/ LMI	Survey	Self-reported	*****	Providing services to mothers, infants and children, prevention and health care
Khodayari-Zarnaq, R. [48]	Iran, 2019/ UMI	Qualitative study	Semi-structured, in-depth qualitative interviews	*****	Establishing and equipping hospitals, supplying medicine and treatment to the poor, financial support for orphans, providing loans to the poor, awareness, training vulnerable groups
Maclure, R. [49]	Burkina Faso, 1995/ LI	Case study	Interview	***	Two NGOs providing first-aid clinics, maternities, and midwife lodgings for providing services for maternal health and child survival
Manna, A. [50]	India, 2019/ LMI	Qualitative study	Interview	**	Using cell phones to communicate with cancer patients
Mehta, P. [51]	India, 2013/ LMI	Cross-sectional	Questionnaire	***	Providing medical services for children with cancers
Momoh, G. T. [52]	Nigeria, 2015/ LMI	Quasi experimental	Semi-structured questionnaire/ in-depth interview	*****	Strengthen the capacity of 12 NGOs in the field of support and policy related to the emphasis on reproductive health issues
Mugisha F [53]	Uganda, 2005/ LI	Qualitative study	Semi-structured individual interviews	*****	Providing reproductive health services
Mukherjee S [54]	India, 2017/ LMI	Descriptive study	Case Studies, semi-structured interviews and unstructured observation/ literature review	*****	Providing community participation
Nguyen, N. [55]	Low- and middle-income countries, 2014	Online survey	Questionnaire	*****	Providing clinical services to cardiovascular programs
Perry, H. [56]	28 countries such as Bangladesh Bolivia Burkina Faso Burundi Cambodia, 2015/ LI, LMI, UMI, HI and Fragile	Qualitative study	N/A	**	Providing maternal, neonatal, and child health services
Perry, H. [57]	28 countries such as Bangladesh Bolivia Burkina Faso Burundi Cambodia, 2015/ LI, LMI, UMI, HI and Fragile	Qualitative study	Review of project Evaluations, presentations at global health conferences, and peer-reviewed publications	***	Providing maternal, neonatal, and child health services
Piotrowicz M [58]	Poland, 2013/ HI	Qualitative study	N/A	*****	
Ricca, J. [59]	Sub-Saharan Africa, South and Southeast Asia and the Caribbean, 2014	Mix method	Structure interview and questioner	*****	NGO projects implementing community-based intervention packages to child mortality
Ui, S. [60]	Cambodia, 2010/ UMI	Descriptive quantitative study	Self-administered questionnaire forms	****	Community participation at health centres in rural
Wu, F. S. [61]	China, 2005/ UMI	Commentary	N/A	N/A	Providing HIV/AIDS prevention
Abdelmoneium, A. O. A. [62]	Sudan, 2010/ LI	Qualitative study	In-depth explanation	*****	Providing for separate provision of services to adolescent mothers
Ahmed, N. [63]	Sudan, 2019/ LI	Qualitative study	In-depth explanation	*****	Providing free vaccinations to children
Kelly, Jeffrey A. [64]	Africa, Central/Eastern Europe and Central Asia, Latin America and the Caribbean, 2006	Qualitative study	In-depth interviews	*****	African NGOs most likely to use peer education and community awareness events; Eastern European NGOs most likely to offer needle exchange; Latin American NGOs to have resource centres and offer risk reduction programmes; and Caribbean organisations to use mass education approaches
Mercer, M. A. [65]	Uganda, India, Brazil, Swaziland, Thailand, Zambia and Kenya, 1991/ LI, LMI, HI	Qualitative study	In-depth explanation	***	Providing educational materials to specific groups, peer education, experimental drugs, counselling and healthcare to people with AIDS
Ambrosini, M. [66]	Italy, 2015/ HI	Case studies	Observation, analysing documents and interviewing	*****	Providing free health care to irregular immigrants
Ament, J. D. [67]	Bolivia, 2014/ LMI	Cross-sectional	Questionnaires/ Interview	*****	Support for spinal procedures
Andrade, M. [68]	Brazil, 2018/ UMI	Non-randomised trial	N/A	*****	Providing diagnostic mammography and biopsies as well as anatomo-histopathological and immunohistochemical analysis
Bader, F. [69]	Jordan, 2009/ UMI	Survey	Interviews	*****	Providing mental health services for displaced Iraqis
Baig, M. B. [70]	India, 2014/ LMI	mixed method	Semi-structured questionnaire/ Interview	*****	Providing primary health centres services
Baqui, A. H. [71]	India, 2008/ LMI	Quasi-experimental study	Questionnaire	*****	Providing maternal and newborn health services
Barzin, Y. [72]	Vietnam, 2012/ LMI	Qualitative study	Interview	*****	Providing health services
Cancedda, C. [73]	Sierra Leone, 2016/ Fragile	Qualitative study	Peer-reviewed publications and after-action reports	****	Provide 17 health facilities in 4 regions and establish two laboratories and employ 800 community health workers to fight Ebola virus disease
Chanani, S. [74]	India, 2019/ LMI	Cross-sectional	Android smartphones and the CommCare mobile application	*****	Providing prevention and treatment services, groups received growth monitoring, referrals to public health facilities, and home-based counselling
Devadasan, N. [75]	India, 2012/ LMI	Cross-sectional survey	interview	*****	Insuring poor people through NGOs
Edward, A. [76]	Afghanistan, 2015/ Fragile	mixed-method	Key informant interviews, focus group discussions / Structured interviews	*****	NGOs provide comprehensive training for community health workers
Ferguson, J. L. [77]	Australia, 2018 / HI	Qualitative study	N/A	***	Providing facilitating diversion from hospitalisation (step-up) and providing residential support services following discharge from the hospital (step down).
Fiorini, G. [78]	Italy, 2016/ HI	Cross-sectional	Anatomical therapeutic chemical	*****	Drug dispensation by a non–governmental organisation providing free medical assistance to undocumented migrants in Milan
Gilbert, H. [79].	Mozambique and Kenya, 2011/ LI And LMI	Qualitative study	N/A	***	Providing HIV/AIDS awareness, HIV/AIDS prevention, access to HIV healthcare services and the provision of treatment
Heinmüller, R. [80]	Mali, 2012/ LI	Time series	Routine data recorded	****	Dispensing free care to under-fives for cases of malaria that covered a rapid diagnostic test and a course of artemisinin-based combination therapy through Medicines Sans Frontiers
Huff-Rousselle, M. [81]	Cambodia, 2001/ LMI	Survey	Questionnaire	****	Providing reproductive health services by an NGO clinic
Mahyiuob Al-Honahi, H. Y. [82]	Yemen, 2010/ Fragile	Descriptive observational study	Pre- and post-intervention surveys	****	Finding and case holding activities by the national TB control programme staff
Matousek, A. C. [83]	Haiti, 2015/ LI	Cross-sectional	Record	***	Providing equitable surgical care in rural Haiti through free care available for the poorest by two NGO hospital
Mukherjee, J. S. [84]	Haiti, 2007/ LI	Mixed method	Structured Interviews/Open-ended questions	***	Zanmi Lasante (NGO) has recruited, trained and financed a large cadre of community health workers to provide such linkages between communities and health centres in rural Haiti
Nunns, D. [85]	Nepal, 2011/ LMI	Country case study	N/A	***	Providing reproductive health care for
Odindo, M. A. [86]	Kenya, 2008/ LMI	Descriptive cross-sectional	Interview	*****	Providing awareness, outreach, counselling, testing, treatment, advocacy, home-based care, assistance to the orphans and legal issues.
Oleribe, O. O. [87]	Nigeria, 2018/ LMI	Cross-sectional	N/A	***	Providing HIV testing and counselling, disclosure of results, post-test counselling and healthy lifestyle education and, distribution of free male condoms and Information, Education and Communication material
Oseji, M [88]	Nigeria, 2014/ LMI	Review	Various publications, reports, public presentations and policy documents	N/A	Providing advocacy, awareness creation, and sensitisation programmes on reproductive health using behaviour change communication materials.
Ridde, V. [89]	Burkina Faso, 2012/ LI	Qualitative study	Interviews and focus group discussions	*****	Providing free HIV treatment and services
Ron, A. [90]	Guatemala and Philippines, 1999/ UMI And LMI	Case two countries study	N/A	***	Providing community health insurance schemes in rural populations
Sankaran, S. [91]	India, 2017/ LMI	Qualitative study	N/A	****	Training community health workers to screen for and manage chronic hypertension
Sarwar, M. R. [92]	Bangladesh, 2015/ LMI	Case studies	N/A	****	Providing maternal and child health and distribution of a micronutrient food supplement
Sharma, A. K [93]	India, 2010/ LMI	Randomised control trial	N/A	****	Providing mid-day meals for primary school students
Singh, M. M [94]	India, 2015/ LMI	Descriptive	Interview	****	Providing care home for PLHA
Singh, V. [95]	India, 2017/ LMI	Quasi-experimental	N/A	****	Providing services delivered by community-based nutrition and health care providers (anganwadi workers and auxiliary nurse midwives)
Sivakumar, T. [96]	India, 2019/ LMI	Cross-sectional descriptive	N/A	****	Providing mental health services through Community Based Rehabilitation
Soe, K. T [97]	Myanmar, 2017/ Fragile	Cross-sectional descriptive	Routine data	*****	Providing community-based TB care to hard-to-reach populations
Solomon, Y. [98]	Mali, 2008/ LI	Mix method	Observation	*****	Providing primary health care
Thomas, R. [99]	India, 2013/ LMI	Cross-sectional descriptive	Routine data	*****	Providing three meals a day
van de Vijver, S. [100]	Kenya, 2013/ LMI	Qualitative study	Previous studies and intervention project and comprehensive literature review	*****	Providing cardiovascular prevention for slums of Nairobi
Wandwalo, E. [101]	Tanzania, 2004/ LMI	Mix method	In-depth interview/ Hospital files and cards, referral forms and laboratory registers	*****	Providing voluntary counselling and testing for HIV, diagnosis and treatment of TB, referral and follow up of patients and suspects, home-based care, psychological support and training
Zachariah, R. [102]	Malawi, 2004/ LMI	Qualitative study	N/A	*****	Providing additional staff, supplementary drugs including antiretroviral drugs, technical assistance and infrastructure development

*TB* tuberculosis, *HIV* human immunodeficiency virus, *AIDS* acquired immune deficiency syndrome, *NGO* non-profit organisation, *LI* low-income, *LMI* low- and middle-income, *UMI* upper-middle-income, *HI* high-income, *PLHA* People Living with HIV/AIDS

### Synthesis of results

**Fig. 2:**
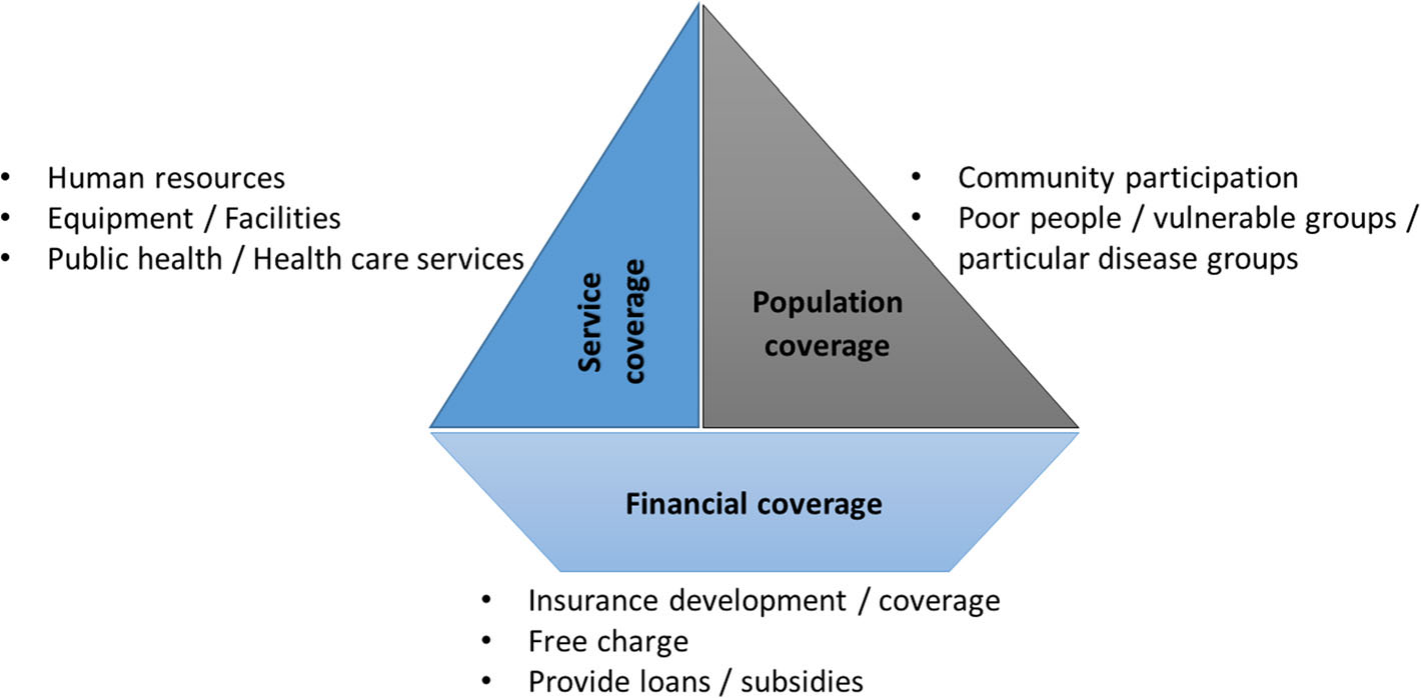
Themes and sub-themes based on the three dimensions of UHC

Based on the three dimensions of the UHC cube (population, service and financial coverage), eight sub-themes related to NGOs’ participation and strategies in achieving UHC were identified (Fig. 2).

### Services coverage

One of the factors influencing NGOs’ participation in achieving UHC is service coverage. NGOs ensured service coverage by providing necessary equipment and health facilities, human resources, and public health and health care services. All 75 studies focused on NGOs’ services coverage [28–102], of which 22 studies were related to human resources, 27 studies focused on health equipment and facilities, and 68 studies related to public health or health care services as strategies for NGOs engagement.

### Population coverage

In many countries, the poor still have limited access to basic health care. NGOs increased population access to health services, particularly for vulnerable and poor or people with a specific disease, by using strategies and interventions through community participation. Seventy-tree studies focused on population coverage [29–102] by NGO’s participation. Out of this, 18 focused on population coverage through community participation, and 69 studies explicitly related to service provision for vulnerable and poor or patients with a specific disease.

### Financial coverage

Protecting people from financial hardship was implemented by supporting them through several strategies, including free or low-cost health care services provision, implementing insurance plans or providing subsidies or loans. Twenty-nine studies focused on the third dimension of UHC, the financial coverage [30–96]. Of these studies, 21 stated that NGOs provided free services, and three reported that NGOs provided insurance plans. Seven studies focused on NGOs offering to subside or provide loans to their covered population. The detailed findings of all included studies are presented in Table 2.

**Table 2:** NGOs participation based on the UHC cube and NGOs financing source

First author (reference no)	NGOs participation base on the UHC cube								NGOs financing source		
	Service coverage			Population coverage		Financial coverage					
	Human resource	Equipment/ Facilities	Public health/ Health care service	Community participation	Poor people/ Need people/ vulnerable groups/ special disease	Insurance development/coverage	Free charge	Providing loans/ subsidies	Government	International organization	Self-financing/ donor subsidies
Yagub, A. I. [28]	*	*	*	*	*					*	
Albis, M. L. F. [29]	*	*	*		*				*		
Amirkhanian, Y. A. [30]		*	*	*	*		*		*	*	*
Bechange, S. [31]	*			*							*
Ejaz, I. [32]	*	*	*		*						
Mercer, M. A. [33]			*		*						
Wamai, R. G [34]		*	*		*			*	*		
Mercer, A [35]		*	*		*		*		*	*	
Mercer, A [36]		*	*		*		*		*	*	
De Maio, G. [37]		*	*		*		*			*	
De Souza, R. [38]			*	*	*						
Dhingra, R. [39]		*	*		*	*					
Franco, M. M. R. [40]		*	*		*			*	*		
Gellert G. A [41]			*	*	*						
Ghosh, S. C. [42]		*			*			*			
Gilson, L. [43]	*	*	*	*	*		*		*	*	*
Gomez-Jauregui, J. [44]			*		*				*	*	
Heard, A [45]	*		*		*		*		*		
Holland, C. E. [46]	*		*	*	*		*				
Khan, J. A. [47]			*		*		*	*			
Khodayari-Zarnaq, R. [48]	*	*	*		*			*	*		
Maclure, R. [49]			*	*	*					*	
Manna, A. [50]	*		*		*		*				
Mehta, P. [51]	*		*		*		*				
Momoh, G. T. [52]	*		*	*	*						
Mugisha F [53]			*		*						
Mukherjee S [54]			*	*							
Nguyen, N. [55]			*		*						
Perry, H. [56]			*	*	*						
Perry, H. [57]			*	*	*						
Piotrowicz M [58]			*				*		*		
Ricca, J. [59]			*	*	*						
Ui, S. [60]		*	*	*	*						
Wu, F. S. [61]	*		*		*						
Abdelmoneium, A. O. A. [62]	*	*	*		*		*		*		
Ahmed, N. [63]			*		*		*		*		
Kelly, Jeffrey A. [64]			*	*	*		*		*	*	*
Mercer, M. A. [65]			*		*						
Ambrosini, M. [66]		*	*	*	*		*				
Ament, J. D. [67]			*		*			*			*
Andrade, M. [68]			*		*						
Bader, F. [69]			*		*						
Baig, M. B. [70]		*	*		*						
Baqui, A. H. [71]			*		*						
Barzin, Y. [72]			*		*		*				
Cancedda, C. [73]	*	*	*		*						
Chanani, S. [74]			*		*		*		*		
Devadasan, N. [75]			*		*	*					
Edward, A. [76]	*			*							
Ferguson, J. L. [77]			*								
Fiorini, G. [78]			*		*		*				
Gilbert, H. [79]			*		*						
Heinmüller, R. [80]	*		*		*		*				
Huff-Rousselle, M. [81]		*	*								
Mahyiuob Al-Honahi, H. Y. [82]			*		*						
Matousek, A. C. [83]		*	*		*		*				
Mukherjee, J. S. [84]	*				*						
Nunns, D. [85]	*	*	*		*					*	
Odindo, M. A. [86]			*		*						
Oleribe, O. O. [87]		*			*						
Oseji, M [88]			*		*						
Ridde, V. [89]			*		*		*			*	
Ron, A. [90]					*	*					
Sankaran, S. [91]	*		*		*						
Sarwar, M. R. [92]		*	*		*						
Sharma, A. K [93]			*		*						
Singh, M. M [94]		*	*		*						
Singh, V. [95]	*		*		*						
Sivakumar, T. [96]			*		*			*			
Soe, K. T [97]	*	*	*		*						
Solomon, Y. [98]	*	*	*	*	*						
Thomas, R. [99]			*		*						
Van de Vijver, S. [100]			*		*						
Wandwalo, E. [101]			*		*						
Zachariah, R. [102]	*	*	*		*						

### Critical appraisal within sources of evidence

The research included 32 qualitative, 29 quantitative, and ten mixed methods studies. Table 3 displays the MMAT quality scores of each included study. Quality appraisal was not done for four papers (three papers were narrative reviews and one paper was a commentary).

**Table 3:** Study design and methodological appraisal scores of included records

MMAT score	20% *	40% **	60% ***	80% ****	100% *****	Total
Study design						
**Qualitative**	**–**	**5**	**8**	**3**	**16**	**32**
**Quantitative**	**–**	**–**	**3**	**10**	**16**	**29**
**Mixed method**	**–**	**–**	**1**	**–**	**9**	**10**
**Total**	**–**	**6**	**13**	**14**	**41**	**71**

## Discussion

We systematically reviewed studies that explored the role of NGOs and their engagement strategies in moving toward UHC. Based on our findings, NGOs have tried to fill in the gaps in health services provision for years. They are increasingly stepping up as healthcare providers, pursuing similar goals, but the government’s inefficiency and resource constraints limit their participation [43]. We discuss our main findings using the UHC cube dimensions.

### Service coverage

NGOs provided qualified personnel for health care services and used a combination of external and internal incentives (including non-financial incentives) to motivate their employees. For example, it was shown that the decision-making, organisational vision, mission and strategy, skills and abilities of NGOs staff positively affect NGOs’ productivity in providing health services [28, 31, 103, 104]. NGOs have improved and promoted the health of their communities through the establishment of primary health centres, laboratory service, training community health workers to screen for and manage chronic hypertension, providing maternal and newborn health services, providing medical services for children with cancers, providing mental health services through community-based rehabilitation, prevention and treatment groups received growth monitoring, referrals to public health facilities, home-based counselling and providing mid-day meals for primary school students and adolescents [34, 51, 70, 71, 74, 91, 93, 95, 96, 99]. For example, in Bangladesh, NGOs provided clinical education, vaccination, reproductive health (antenatal and postnatal care, skilled birth attendance, breastfeeding prevalence, contraceptive prevalence, sexually transmitted infections), child and infants health services (child diarrhoea), acute respiratory infection and HIV/AIDS awareness [29, 35, 36, 47]. In India, an NGO was contracted to deliver basic health services, including simple curative care, referral for more complex cases, identification and registration of pregnant women, perinatal care, referral for a complicated pregnancy or high-risk births, essential child health care, assistance with immunisation and other national programmes, and the conduct of health camps for outreach and health education provided [45].

Many NGOs offer a wide range of HIV/AIDS-related services. For example, in Central and Eastern Europe, NGO programs often targeted injecting drug users, and activities included needle exchange, HIV prevention education, services for people with AIDS, and the distribution of educational materials [30, 64]. In Uganda, NGOs provided health services such as educational materials, peer education, experimental drugs, counselling and healthcare to people with AIDS [31, 65]. In Ethiopia, Kenya and Mozambique, NGOs provided HIV/AIDS health services in clinics, raised HIV/AIDS awareness, participated in HIV/AIDS prevention, ensured access to HIV healthcare services, and provided treatment [34, 79]. NGOs provided clinical and family planning services in India, organised health awareness camps, and campaigned for immunisation and HIV/AIDS awareness [105].

### Population coverage

In many countries, the poor still have limited access to essential health care, and NGOs are increasing access to health services because of their ability to design population-based projects. NGOs are also in a position to implement prevention programs with the potential to reach vulnerable social populations, and the use of innovative approaches such as the caregiver approach can be a promising alternative to existing strategies to provide critical health care to disadvantaged communities [29, 30, 32, 56]. NGOs have sought to fill a gap in the Pakistani public sector due to a lack of healthcare providers’ access. East Timor and Sudan are examples of post-crisis countries in which NGOs efforts in the first place to assist the affected people were vital. In Bangladesh, NGO partnerships with the government have resulted in relatively high coverage for reproductive and child health services and reduced infant and child mortality. The main focus of NGOs in Italy was mainly on the homeless and immigrants, including immigrant and indigenous homeless, irregular immigrants, undocumented migrants and migrant populations with HIV/AIDS [28, 32, 33, 35, 37, 66, 78, 106].

Community mobilisation is a factor that increases the positive impact of prevention programs, such as HIV. NGOs that manage health care facilities and created health care projects made significant efforts to involve the community in providing health care. NGOs involved members of at-risk communities in the social activities of the community [28, 30]. Using social networks to reach men who have sex with men, they connected more significant numbers of the population to effective HIV interventions, which will improve health outcomes and the success of Ugandan AIDS/HIV NGOs public projects that largely depends on the NGO network [31, 46].

Overall, NGOs’ strengths can be found in their desire to provide quality service and service in relatively remote areas. Establishing NGOs in LMICs with complex medical procedures, such as hematopoietic stem-cell transplantation, is also essential for disadvantaged populations [40, 107]. Even those without health expertise and limited resources can effectively promote and facilitate community participation in health centre management. Such NGOs’ roles are critically important for sustainable health development and should be further recognised and supported [60]. Strengthening community capacity as community mobilisation can help increase awareness, demand and utilisation of health services and local people’s involvement in project planning, implementation, and evaluation [31, 54].

### Financial coverage

UHC is a key priority set out by the WHO and the United Nations General Assembly [108, 109]. Social health insurance (SHI) schemes, one mechanism to achieve UHC, has become increasingly crucial in LMICs as they work to achieve this goal. To ensure comprehensive health insurance coverage for a broad population at a reasonable cost, SHI schemes are generally designed so that individuals pay into a central fund, either indirectly through taxes or directly through wage-based contributions, and receive a set package of subsidised health services through accredited providers [108, 110, 111]. However, because specific populations cannot afford any financial contribution through taxes or direct payments, many countries created hybrid SHI systems in which government funds cover these population groups [112].

Reducing health costs is one of the most critical dimensions of UHC, and NGOs can reduce the poor’s financial burden through their programs [40, 50]. For example, the participation of NGOs in Indian health insurance schemes stems from the following reasons. First, in India, private spending is about two-thirds of health care costs. Second, the quality of available health care for the Indian people is poor. Third, India’s health insurance coverage is limited, especially among those working outside the formal sector [39]. Using new strategies (e.g., telephone communication in palliative care) can reduce patients’ financial problems. NGOs can help patients through drug subsidies and chemotherapy in very costly diseases such as hematopoietic stem-cell transplantation. In India, an NGO organised all community health insurance (CHI) schemes which increased access to health care and reduced out-of-pocket payments [75]. In Sudan, NGOs provided free vaccinations to children. In Bangladesh, NGOs established accessible toilets or provided loans in rural areas to improve their health. In Italy, NGOs providing free health care to irregular immigrants [42, 63, 66].

Overall, NGOs need sustained financial support to implement their plans and programs. A decentralised approach to a country’s political structure can lead to NGOs’ financial stability and productive cooperation between NGOs and the government [44, 53]. NGOs can provide financial resources themselves using a variety of strategies, including international and charitable foundations, international aid organisations, local government, foreign governments, business activity, tax exemptions, tax subsidies, and donors [28, 30, 31, 33, 40, 43–46, 48, 53, 113, 114]. In countries where HIV/AIDS prevention NGOs are active, but extensive national government financial support is lacking, NGOs’ secured funding and resources enabled access to prevention, treatment, and improved HIV care [30, 46]. However, not all NGOs successfully reach their goals, especially when they face a two-track problem of building efficient service delivery to meet current needs and taking on the long-term state-building tasks that assist in establishing durable, local delivery systems, especially when such mechanisms are lacking [115].

### Limitations

Due to the subject’s extent, our study was limited to published papers only and excluded grey literature, which could limit our review’s scope. Nonetheless, we accessed the quality of published studies and included all types of studies (i.e., qualitative, quantitative, and mixed-methods), which allowed for a comprehensive overview.

## Conclusion

Significant reforms in the health system are needed to achieve UHC, but governments cannot do it alone. Accounting for possible strengths and capabilities of NGOs and sharing their resources is a potential way to reach UHC. Despite the critical role of NGOs in health services delivery, relatively little is still known about how they can engage these organisations to achieve UHC goals. NGOs could play a pivotal role in moving towards UHC alongside the government and other groups or organisations. Understanding NGOs’ role and contributing to attaining UHC is critical, especially in the local context. Governments need to consider systematic and fundamental strategies for engaging NGOs towards public health goals to move towards UHC. Given the creation and expansion of health services and global attention to UHC, NGOs’ presence can improve financial support and improve the status of services provided to the poor and marginalised areas.

## Supplementary Information

**Additional file 1.** Appendix 1: Preferred Reporting Items for Systematic reviews and Meta-Analyses extension for Scoping Reviews (PRISMA-ScR) Checklist. Appendix 2: Full search strategy with results. Appendix3: Quality assessment of selected studies.

## Data Availability

The data are openly available upon request from the corresponding author.
