# Supplementary material for: Engagement of non-governmental organisations in moving towards universal health coverage: a scoping review
Source: Global Health. 2021 Nov 16;17:129. doi: 10.1186/s12992-021-00778-1 (PMC8594189; doi:10.1186/s12992-021-00778-1)
Supplement: Supplementary file 1 — Additional file 1. Appendix 1: Preferred Reporting Items for Systematic reviews and Meta-Analyses extension for Scoping Reviews (PRISMA-ScR) Checklist. Appendix 2: Full search strategy with results. Appendix3: Quality assessment of selected studies. [file 12992_2021_778_MOESM1_ESM.docx]

**SUPPLEMENTARY MATERIAL**

**APPENDIX 1: Preferred Reporting Items for Systematic reviews and Meta-Analyses extension for Scoping Reviews (PRISMA-ScR) Checklist**

| **SECTION** | **ITEM** | **PRISMA-ScR CHECKLIST ITEM** | **REPORTED ON PAGE #** |
| --- | --- | --- | --- |
| **TITLE** | | | |
| Title | 1 | Identify the report as a scoping review. | 1 |
| **ABSTRACT** | | | |
| Structured summary | 2 | Provide a structured summary that includes (as applicable): background, objectives, eligibility criteria, sources of evidence, charting methods, results, and conclusions that relate to the review questions and objectives. | 1 |
| **INTRODUCTION** | | | |
| Rationale | 3 | Describe the rationale for the review in the context of what is already known. Explain why the review questions/objectives lend themselves to a scoping review approach. | 2 |
| Objectives | 4 | Provide an explicit statement of the questions and objectives being addressed with reference to their key elements (e.g., population or participants, concepts, and context) or other relevant key elements used to conceptualize the review questions and/or objectives. | 2 |
| **METHODS** | | | |
| Protocol and registration | 5 | Indicate whether a review protocol exists; state if and where it can be accessed (e.g., a Web address); and if available, provide registration information, including the registration number. | 3 |
| Eligibility criteria | 6 | Specify characteristics of the sources of evidence used as eligibility criteria (e.g., years considered, language, and publication status), and provide a rationale. | 3 |
| Information sources* | 7 | Describe all information sources in the search (e.g., databases with dates of coverage and contact with authors to identify additional sources), as well as the date the most recent search was executed. | 3 |
| Search | 8 | Present the full electronic search strategy for at least 1 database, including any limits used, such that it could be repeated. | 3 |
| Selection of sources of evidence† | 9 | State the process for selecting sources of evidence (i.e., screening and eligibility) included in the scoping review. | 3 |
| Data charting process‡ | 10 | Describe the methods of charting data from the included sources of evidence (e.g., calibrated forms or forms that have been tested by the team before their use, and whether data charting was done independently or in duplicate) and any processes for obtaining and confirming data from investigators. | 3 |
| Data items | 11 | List and define all variables for which data were sought and any assumptions and simplifications made. | 3 |
| Critical appraisal of individual sources of evidence§ | 12 | If done, provide a rationale for conducting a critical appraisal of included sources of evidence; describe the methods used and how this information was used in any data synthesis (if appropriate). | 3 |
| Synthesis of results | 13 | Describe the methods of handling and summarizing the data that were charted. | 3 |
| **RESULTS** | | | |
| Selection of sources of evidence | 14 | Give numbers of sources of evidence screened, assessed for eligibility, and included in the review, with reasons for exclusions at each stage, ideally using a flow diagram. | 4 |
| Characteristics of sources of evidence | 15 | For each source of evidence, present characteristics for which data were charted and provide the citations. | 4 |
| Critical appraisal within sources of evidence | 16 | If done, present data on critical appraisal of included sources of evidence (see item 12). | 5 |
| Results of individual sources of evidence | 17 | For each included source of evidence, present the relevant data that were charted that relate to the review questions and objectives. | 4,5 |
| Synthesis of results | 18 | Summarize and/or present the charting results as they relate to the review questions and objectives. | 4,5 |
| **DISCUSSION** | | | |
| Summary of evidence | 19 | Summarize the main results (including an overview of concepts, themes, and types of evidence available), link to the review questions and objectives, and consider the relevance to key groups. | 5-7 |
| Limitations | 20 | Discuss the limitations of the scoping review process. | 7 |
| Conclusions | 21 | Provide a general interpretation of the results with respect to the review questions and objectives, as well as potential implications and/or next steps. | 8 |
| **FUNDING** | | | |
| Funding | 22 | Describe sources of funding for the included sources of evidence, as well as sources of funding for the scoping review. Describe the role of the funders of the scoping review. | 8 |

**SUPPLEMENTARY MATERIAL**

**APPENDIX 2: FULL SEARCH STRATEGY WITH RESULTS**

**PubMed: August 30, 2020**

|  | Search term | Hints |
| --- | --- | --- |
| #1 | Search "health service"[Title/Abstract] OR "health system"[Title/Abstract] OR "health service delivery"[Title/Abstract] OR "service coverage"[Title/Abstract] OR "universal health coverage"[Title/Abstract] OR "population coverage"[Title/Abstract] OR "financial coverage"[Title/Abstract] OR "financial"[Title/Abstract] OR "UHC"[Title/Abstract] OR "care service"[Title/Abstract] | 174,361 |
| #2 | Search "NGO"[Title/Abstract] OR "NGOs"[Title/Abstract] OR "non-governmental organization"[Title/Abstract] OR "non-state providers"[Title/Abstract] | 3,909 |
| #3 | Search ((("NGO"[Title/Abstract] OR "NGOs"[Title/Abstract]) OR "non-governmental organization"[Title/Abstract]) OR "non-state providers"[Title/Abstract]) AND ((((((((("health service"[Title/Abstract] OR "health system"[Title/Abstract]) OR "health service delivery"[Title/Abstract]) OR "service coverage"[Title/Abstract]) OR "universal health coverage"[Title/Abstract]) OR "population coverage"[Title/Abstract]) OR "financial coverage"[Title/Abstract]) OR "financial"[Title/Abstract]) OR "UHC"[Title/Abstract]) OR "care service"[Title/Abstract]) | 609 |

**Scopus**: **August 30, 2020**

|  | **Search terms** | **Hints** |
| --- | --- | --- |
| #1 | TITLE-ABS-KEY ( ngo  OR  ngos  OR  "non-governmental organization"  OR  "non-state providers" ) | 31,880 |
| #2 | TITLE-ABS-KEY ( "health service"  OR  "health system"  OR  "health service delivery"  OR  "service coverage"  OR  "universal health coverage"  OR  "population coverage"  OR  "financial coverage"  OR  financial  OR  uhc  OR  "care service" ) | 1,362,892 |
| #3 | ( TITLE-ABS-KEY ( ngo  OR  ngos  OR  "non-governmental organization"  OR  "non-state providers" ) )  AND  ( TITLE-ABS-KEY ( "health service"  OR  "health system"  OR  "health service delivery"  OR  "service coverage"  OR  "universal health coverage"  OR  "population coverage"  OR  "financial coverage"  OR  financial  OR  uhc  OR  "care service" ) ) | 4,317 |

**ProQuest**: **August 30, 2020**

|  | **Search terms** | **Hints** |
| --- | --- | --- |
| #1 | ab(NGO OR NGOs OR "non-governmental organization" OR "non-state providers") | 3,017 |
| #2 | ab("health service" OR "health system" OR "health service delivery" OR "service coverage" OR "universal health coverage" OR "population coverage" OR "financial coverage" OR financial OR UHC OR "care service") | 96,720 |
| #3 | ab(NGO OR NGOs OR "non-governmental organization" OR "non-state providers") AND ab("health service" OR "health system" OR "health service delivery" OR "service coverage" OR "universal health coverage" OR "population coverage" OR "financial coverage" OR financial OR UHC OR "care service") | 354 |

**Web of Science: August 30, 2020**

|  | **Search term** | **Hints** |
| --- | --- | --- |
| #1 | TS=(NGO OR  NGOs  OR  “non-governmental  organization”  OR  “non-state  providers”) | 17,311 |
| #2 | TS=("health service" OR "health system" OR "health service delivery" OR "service coverage" OR "universal health coverage" OR "population coverage" OR "financial coverage" OR financial OR UHC OR "care service") | 389,491 |
| #3 | #1 AND #2 | 1,334 |

**EMBASE: August 30, 2020:**

|  | **Search terms** | **Hints** |
| --- | --- | --- |
| #1 | ngo:ti,ab,kw OR ngos:ti,ab,kw OR 'non-governmental organization':ti,ab,kw OR 'non-state providers':ti,ab,kw | 5,710 |
| #2 | 'health service':ti,ab,kw OR 'health system':ti,ab,kw OR 'health service delivery':ti,ab,kw OR 'service coverage':ti,ab,kw OR 'universal health coverage':ti,ab,kw OR 'population coverage':ti,ab,kw OR 'financial coverage':ti,ab,kw OR financial:ti,ab,kw OR uhc:ti,ab,kw OR 'care service':ti,ab,kw | 233,612 |
| #3 | #1 AND #2 | 836 |

**SUPPLEMENTARY MATERIAL**

**Appendix3: Quality assessment of selected studies**

**Introduction:**

For each included article, the total quality score may not contain useful data (in comparison with a descriptive summary through using MMAT criterion); however, it is possible to calculate the score using MMAT. Since there are only a few criteria for each domain, the score can be represented through descriptors like *, **, ***, **** and *****. For included studies, this score can be the number of the criteria met, which is divided into five subcategories (categories from 20% (*) -one criterion met- to 100% (*****) -all the criteria met-).

**Table A1 Quality assessment for qualitative studies**

| **First author  (Reference No)** | **1 - Are there clear research questions?** | **2 - Do the collected data allow to address the research questions?** | **3- Is the qualitative approach appropriate to answer the research question?** | **4 - Are the qualitative data collection methods adequate to address the research question?** | **5 - Are the findings adequately derived from the data?** | **6 - Is the interpretation of results sufficiently substantiated by data?** | **7 - Is there coherence between qualitative data sources, collection, analysis and interpretation?** | **Score** |
| --- | --- | --- | --- | --- | --- | --- | --- | --- |
| ‏ Amirkhanian YA, ^1^ | Yes | Yes | Yes | Yes | Yes | Yes | Yes | ***** |
| Ejaz I, ^2^ | Yes | Yes | Yes | Yes | Yes | Yes | Yes | ***** |
| Mercer MA, ^3^ | Yes | Yes | Can’t tell | Can’t tell | Yes | Yes | Can’t tell | ** |
| Wamai RG, ^4^ | Yes | Yes | Can’t tell | Can’t tell | Yes | Yes | Yes | *** |
| de Souza R, ^5^ | Yes | Yes | Yes | Yes | Yes | Yes | Yes | ***** |
| Dhingra R, ^6^ | Yes | Yes | Can’t tell | Can’t tell | Yes | Yes | Can’t tell | ** |
| Gellert GA ‏, ^7^ | Yes | Yes | Can’t tell | Can’t tell | Yes | Yes | Can’t tell | ** |
| Gomez-Jauregui J, ^8^ | Yes | Yes | Yes | Yes | Yes | Yes | Yes | ***** |
| Khodayari-Zarnaq R, ^9^ | Yes | Yes | Yes | Yes | Yes | Yes | Yes | ***** |
| Maclure R, ^10^ | Yes | Yes | Can’t tell | Can’t tell | Yes | Yes | Yes | *** |
| Manna A, ^11^ | Yes | Yes | Can’t tell | Can’t tell | Yes | Yes | Can’t tell | ** |
| Mugisha F, ^12^ | Yes | Yes | Yes | Yes | Yes | Yes | Yes | ***** |
| Mukherjee S, ^13^ | Yes | Yes | Yes | Yes | Yes | Yes | Yes | ***** |
| Perry H, ^14^ | Yes | Yes | Can’t tell | Can’t tell | Yes | Yes | Can’t tell | ** |
| Perry H, ^15^ | Yes | Yes | Can’t tell | Can’t tell | Yes | Yes | Yes | *** |
| Abdelmoneium AOA ‏, ^16^ | Yes | Yes | Yes | Yes | Yes | Yes | Yes | ***** |
| Ahmed N ‏, ^17^ | Yes | Yes | Yes | Yes | Yes | Yes | Yes | ***** |
| Kelly JA, ^18^ | Yes | Yes | Yes | Yes | Yes | Yes | Yes | ***** |
| Mercer MA, ^19^ | Yes | Yes | Can’t tell | Can’t tell | Yes | Yes | Yes | *** |
| Ambrosini M, ^20^ | Yes | Yes | Yes | Yes | Yes | Yes | Yes | ***** |
| Barzin Y, ^21^ | Yes | Yes | Yes | Yes | Yes | Yes | Yes | ***** |
| Cancedda C, ^22^ | Yes | Yes | Can’t tell | Yes | Yes | Yes | Yes | **** |
| Piotrowicz M, ^23^ | Yes | Yes | Yes | Yes | Yes | Yes | Yes | ***** |
| Ferguson JL, ^24^ | Yes | Yes | Can’t tell | Can’t tell | Yes | Yes | Yes | *** |
| Gilbert H, ^25^ | Yes | Yes | Can’t tell | Can’t tell | Yes | Yes | Yes | *** |
| Nunns D, ^26^ | Yes | Yes | Yes | Can’t tell | Can’t tell | Yes | Yes | *** |
| Ridde V, ^27^ | Yes | Yes | Yes | Yes | Yes | Yes | Yes | ***** |
| Ron A, ^28^ | Yes | Yes | Can’t tell | Yes | Can’t tell | Yes | Yes | *** |
| Sankaran S, ^29^ | Yes | Yes | Yes | Yes | Can’t tell | Yes | Yes | **** |
| Sarwar MR ‏, ^30^ | Yes | Yes | Yes | Yes | Can’t tell | Yes | Yes | **** |
| van de Vijver S, ^31^ | Yes | Yes | Yes | Yes | Yes | Yes | Yes | ***** |
| Zachariah R, ^32^ | Yes | Yes | Yes | Yes | Yes | Yes | Yes | ***** |

**Table A2 Quality assessment for quantitative descriptive studies**

| **Title**  **(Reference No)** | **1 - Are there clear research questions?** | **2 - Do the collected data allow to address the research questions?** | **3 - Is the sampling strategy relevant to address the research question?** | **4 - Is the sample representative of the target population?** | **5 - Are the measurements appropriate?** | **6 - Is the risk of nonresponse bias low?** | **7 - Is the statistical analysis appropriate to answer the research question?** | **Total score** |
| --- | --- | --- | --- | --- | --- | --- | --- | --- |
| Mercer A, ^33^ | Yes | Yes | Yes | Yes | Yes | Can’t tell | Yes | **** |
| Ui S ^34^ | Yes | Yes | Yes | Yes | Yes | Can’t tell | Yes | **** |
| Albis MLF, ^35^ | Yes | Yes | Yes | Yes | Yes | Can’t tell | Yes | **** |
| De Maio G, ^36^ | Yes | Yes | Yes | Yes | Yes | Can’t tell | Yes | **** |
| Nguyen N, ^37^ | Yes | Yes | Yes | Yes | Yes | Yes | Yes | ***** |
| Bader F ‏, ^38^ | Yes | Yes | Yes | Yes | Yes | Yes | Yes | ***** |
| Odindo MA, ^39^ | Yes | Yes | Yes | Yes | Yes | Yes | Yes | ***** |
| Oleribe OO, ^40^ | Yes | Yes | Yes | Can’t tell | Can’t tell | Yes | Yes | *** |
| Singh MM ‏, ^41^ | Yes | Yes | Yes | Yes | Yes | Can’t tell | Yes | **** |
| Sivakumar T, ^42^ | Yes | Yes | Yes | Yes | Yes | Can’t tell | Yes | **** |
| Soe KT, ^43^ | Yes | Yes | Yes | Yes | Yes | Yes | Yes | ***** |
| Thomas R, ^44^ | Yes | Yes | Yes | Yes | Yes | Yes | Yes | ***** |

**Table A3 Quality assessment for Quantitative non- randomized**

| **Title  (Reference No)** | **1 - Are there clear research questions?** | **2 - Do the collected data allow to address the research questions?** | **3 - Are the participant’s representative of the target population?** | **4 - Are measurements appropriate regarding both the outcome and intervention (or exposure)?** | **5 - Are there complete outcome data?** | **6 - Are the confounders accounted for in the design and analysis?** | **7 - During the study period, is the intervention administered (or exposure occurred) as intended?** | **Total score** |
| --- | --- | --- | --- | --- | --- | --- | --- | --- |
| Franco MMR, ^45^ | Yes | Yes | Yes | Yes | Yes | Yes | Yes | ***** |
| Holland CE, ^46^ | Yes | Yes | Yes | Yes | Yes | Yes | Yes | ***** |
| Khan JA, ^47^ | Yes | Yes | Yes | Yes | Yes | Yes | Yes | ***** |
| Mehta P, ^48^ | Yes | Yes | Yes | Can’t tell | Yes | Can’t tell | Yes | *** |
| Momoh GT, ^49^ | Yes | Yes | Yes | Yes | Yes | Yes | Yes | ***** |
| Ament JD, ^50^ | Yes | Yes | Yes | Yes | Yes | Yes | Yes | ***** |
| Andrade M, ^51^ | Yes | Yes | Yes | Yes | Yes | Yes | Yes | ***** |
| Baqui AH ‏, ^52^ | Yes | Yes | Yes | Yes | Yes | Yes | Yes | ***** |
| ‏ Chanani S, ^53^ | Yes | Yes | Yes | Yes | Yes | Yes | Yes | ***** |
| Devadasan N, ^54^ | Yes | Yes | Yes | Yes | Yes | Yes | Yes | ***** |
| Fiorini G, ^55^ | Yes | Yes | Yes | Yes | Yes | Yes | Yes | ***** |
| Heinmüller R, ^56^ | Yes | Yes | Yes | Yes | Can’t tell | Yes | Yes | **** |
| Huff-Rousselle M, ^57^ | Yes | Yes | Yes | Yes | Can’t tell | Yes | Yes | **** |
| Mahyiuob Al-Honahi HY, ^58^ | Yes | Yes | Yes | Can’t tell | Yes | Yes | Yes | **** |
| ‏ Matousek AC,^59^ | Yes | Yes | Yes | Can’t tell | Can’t tell | Yes | Yes | *** |

**Table A4 Quality assessment for mixed methods studies**

| **Title  (Reference No)** | **1 - Are there clear research questions?** | **2 - Do the collected data allow to address the research questions?** | **3 - Is there an adequate rationale for using a mixed methods design to address the research question?** | **4 - Are the different components of the study effectively integrated to answer the research question?** | **5 - Are the outputs of the integration of qualitative and quantitative components adequately interpreted?** | **6 - Are divergences and inconsistencies between quantitative and qualitative results adequately addressed?** | **7 - Do the different components of the study adhere to the quality criteria of each tradition of the methods involved?** | **Total score** |
| --- | --- | --- | --- | --- | --- | --- | --- | --- |
| Yagub AI, ^60^ | Yes | Yes | Yes | Yes | Yes | Yes | Yes | ***** |
| Ricca J, ^61^ | Yes | Yes | Yes | Yes | Yes | Yes | Yes | ***** |
| Bechange S, ^62^ | Yes | Yes | Yes | Yes | Yes | Yes | Yes | ***** |
| Ghosh SC, ^63^ | Yes | Yes | Yes | Yes | Yes | Yes | Yes | ***** |
| Heard A, ^64^ | Yes | Yes | Yes | Yes | Yes | Yes | Yes | ***** |
| Baig MB, ^65^ | Yes | Yes | Yes | Yes | Yes | Yes | Yes | ***** |
| Edward A, ^66^ | Yes | Yes | Yes | Yes | Yes | Yes | Yes | ***** |
| Mukherjee JS, ^67^ | Yes | Yes | Yes | Yes | Can’t tell | Can’t tell | Yes | *** |
| Solomon Y, ^68^ | Yes | Yes | Yes | Yes | Yes | Yes | Yes | ***** |
| Wandwalo E, ^69^ | Yes | Yes | Yes | Yes | Yes | Yes | Yes | ***** |

**Table A5 Quality assessment for randomized control trial**

| **Title**  **(Reference No)** | **1 - Are there clear research questions?** | **2 - Do the collected data allow to address the research questions?** | **3 - Is randomization appropriately performed?** | **4 - Are the groups comparable at baseline?** | **5 - Are there complete outcome data?** | **6 - Are outcome assessors blinded to the intervention provided?** | **7 - Did the participants adhere to the assigned intervention?** | **Total score** |
| --- | --- | --- | --- | --- | --- | --- | --- | --- |
| Sharma AK, ^70^ | Yes | Yes | Yes | Yes | Yes | Can’t tell | Yes | **** |
| Singh V, ^71^ | Yes | Yes | Yes | Yes | Yes | Yes | Yes | ***** |
